# Data-driven schizophrenia subtyping via brain atrophy trajectories and functional connectivity

**DOI:** 10.1038/s41398-026-03968-w

**Published:** 2026-03-19

**Authors:** Daisuke Yoshimaru, Kazuya Ouchi, Shuhei Shibukawa, Masakazu Ozawa, Motoki Hirabayashi, Ami Yuzawa, Tomokazu Tsurugizawa, Kenichi Oishi, Hirotaka James Okano

**Affiliations:** 1 Division of Regenerative Medicine, The Jikei University School of Medicine, Tokyo, 105-8461 Japan; 2 National Institute of Advanced Industrial Science and Technology (AIST), Tsukuba, 305-8565 Japan; 3 Department of Radiology, Tokyo Medical University, Tokyo, 160-0023 Japan; 4 Faculty of Engineering, University of Tsukuba, Tsukuba, Ibaraki, 305-8573 Japan; 5 Department of Radiological Technology, Faculty of Health Science, Juntendo University, Tokyo, 113-8421 Japan; 6 Center for Evolutionary Cognitive Sciences, Graduate School of Arts and Sciences, The University of Tokyo, Tokyo, 153-8902 Japan; 7 Department of Neurology, The Jikei University School of Medicine, Tokyo, 105-8461 Japan; 8 Department of Otorhinolaryngology, The Jikei University School of Medicine, Tokyo, 105-8461 Japan; 9 Department of Radiology, The Johns Hopkins University School of Medicine, Baltimore, MD 21205 USA; 10 Department of Neurology, The Johns Hopkins University School of Medicine, Baltimore, MD 21205 USA

**Keywords:** Predictive markers, Schizophrenia

## Abstract

Schizophrenia exhibits substantial clinical and neuroanatomical heterogeneity, yet conventional cross-sectional studies typically analyse patients at different disease stages as homogeneous groups, diluting pathological diversity. We applied the Subtype and Stage Inference (SuStaIn) algorithm to structural MRI data from 85 patients with schizophrenia and 224 healthy controls, integrating this with resting-state functional MRI analysis for the first time. SuStaIn identified two distinct neuroanatomical subtypes: Subtype0 demonstrated anterior-to-posterior progression with early frontal-limbic atrophy and prominent positive symptoms, while Subtype1 exhibited posterior-to-anterior progression originating in subcortical-occipital regions with greater social withdrawal. Disease progression stage correlated with functional connectivity in opposing directions—Subtype0 showed hypoconnectivity (ρ = −0.56) while Subtype1 exhibited hyperconnectivity (ρ = 0.70). These contrasting patterns may explain decades of contradictory functional connectivity findings in schizophrenia literature, as previous studies likely captured different subtypes at various disease stages. Our integrated structure-function approach reveals distinct disease trajectories that could inform subtype-specific therapeutic strategies.

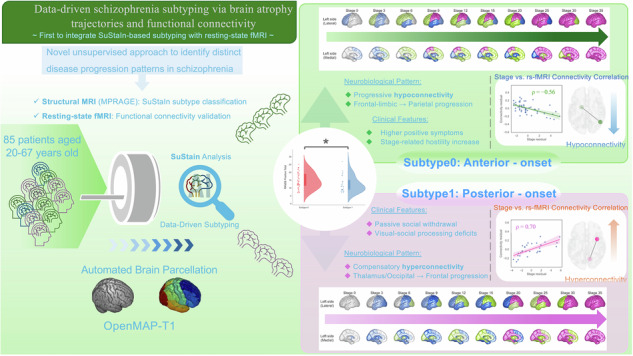

## Introduction

Schizophrenia is a highly heterogeneous psychiatric disorder characterised by substantial individual differences in clinical symptoms, cognitive function, and treatment responsiveness [1]. Understanding pathophysiological substrates that cause diversity in clinical characteristics is essential for implementing precision medicine. However, traditional symptom-based classifications have proven inadequate for identifying true subtypes grounded in pathophysiological mechanisms [2–4].

A fundamental limitation of schizophrenia subtype research is the absence of classification systems that incorporate temporal disease progression. Conventional cross-sectional studies often analyse patients at different disease stages as homogeneous groups, thereby diluting pathological variability and complicating efforts to obtain consistent findings. To address this limitation, dynamic subtype classification using the Subtype and Stage Inference (SuStaIn) algorithm [5] has recently gained traction.

Recent large-scale SuStaIn-based studies have marked significant advances in schizophrenia research. Jiang et al. [6] conducted an international multicentre study of 4291 patients, identifying two distinct subtypes using SuStaIn: an early cortical-predominant loss subtype originating from Broca’s area and the anterior insular cortex, and an early subcortical-predominant loss subtype originating in the hippocampus. Sone et al. [7] further demonstrated clinical utility by showing that treatment-resistant patients exhibited significantly more advanced disease stages.

While these studies established structural subtypes based on atrophy patterns, important limitations remain. A major issue is the lack of integration between structural and functional changes in the brain. Current research has relied solely on structural MRI data without exploring functional connectivity relationships. This gap hinders unified interpretation of the long-standing debate hypoconnectivity versus hyperconnectivity in schizophrenia functional MRI (fMRI) research [8–13]. Leveraging SuStaIn-derived disease progression stage information in fMRI analysis could quantify connectivity changes across disease stages, potentially identifying optimal time points for therapeutic intervention.

This study integrated SuStaIn-based structural subtype classification with resting-state fMRI analysis for the first time to comprehensively characterise both structural and functional changes in schizophrenia subtypes. By investigating correlations between disease progression stages and functional connectivity, we aimed to provide new insights into the hypo- and hyperconnectivity patterns observed in schizophrenia and establish a foundation for developing subtype-specific therapeutic strategies.

## Materials and methods

### MRI data acquisition

The data used in this study were obtained from the DecNef Project Brain Data Repository (https://bicr-resource.atr.jp/srpbsopen/), a multi-site, multi-disorder resting-state MRI database established by a research consortium under the Japanese Strategic Research Program for the Promotion of Brain Science (SRPBS), supported by the Advanced Research and Development Programs for Medical Innovation (a program under the Japan Agency for Medical Research and Development [AMED]). All participants in the original studies provided written informed consent in accordance with the Declaration of Helsinki, with approval from the Committee on Medical Ethics of Kyoto University. The present study involved secondary analyses of de-identified data; no additional ethical approval was required at our institution. The present study involved secondary analyses of de-identified data; no additional ethical approval was required at our institution. Sample size was determined by data availability rather than a priori power calculations. The final sample represents all participants meeting inclusion criteria from this database. For this analysis, we utilised single-site T1-weighted three-dimensional structural MRI and resting-state fMRI data from this dataset. All scans were acquired using one of two Siemens 3.0 Tesla systems (MAGNETOM Trio vs. MAGNETOM Trio A Tim System, Erlangen, Germany). Both systems employed identical core hardware specifications for neuroimaging, including the same OR64 superconducting magnet and XQ gradient set (45 mT/m, 200 T/m/s). The Tim upgrade added only higher-channel radiofrequency hardware without altering the gradient or shim performance, ensuring consistency in neuroimaging capabilities across both systems. For structural T1-weighted magnetisation-prepared rapid gradient-echo sequences, the acquisition parameters were highly standardised and nearly identical between the scanners (see Supplementary Table S1). Given the minimal technical differences between systems, no harmonisation procedures were applied to the structural data. fMRI data were acquired using echo-planar imaging sequences with different protocols between the scanners. The MAGNETOM Trio used TR/TE = 2000/30 ms with 4 × 4 × 4 mm voxels, while the MAGNETOM Trio A Tim System used TR/TE = 2500/30 ms with 3.3 × 3.3 × 3.2 mm voxels. These technical differences were systematically evaluated and accounted for through statistical modelling by including scanner type as a covariate in all group-level analyses alongside age, sex, and handedness. Scanner-specific effects were tested for all reported connections using one-way ANOVA (see Supplementary Table S1).

### Image processing and brain parcellation

Brain parcellation was performed using *OpenMAP-T1* (https://github.com/OishiLab/OpenMAP-T1), a deep learning-based framework that enables rapid, high-resolution whole-brain segmentation into 280 anatomical regions based on the Johns Hopkins University–Montreal Neurological Institute (JHU-MNI) atlas [14, 15] (see Supplementary Methods S2 for details on preprocessing and parcellation procedures).

### Region selection and volume extraction

For the present analysis, we focused on a subset of brain regions previously implicated in schizophrenia. These included cortical regions (frontal, temporal, parietal, and occipital lobes, analysed separately for the left and right hemispheres) and subcortical structures (limbic regions, insula, amygdala, basal ganglia, and thalamus). Detailed definitions of all selected regions are provided in Supplementary Table S3.

For cortical regions, the volumes of the left and right hemispheres were analysed separately to assess potential asymmetries in atrophy patterns. For subcortical structures, the volumes from the left and right hemispheres were averaged to reduce dimensionality and increase statistical power while preserving clinically meaningful patterns. Regional volumes were adjusted for age, sex, and total intracranial volume (TIV) using linear regression in the control group, and residuals were normalized to z-scores for SuStaIn analysis (see Supplementary Methods S4 for details). TIV was calculated as the sum of grey matter, white matter, and cerebrospinal fluid volumes.

### SuStaIn analysis

SuStaIn is a probabilistic machine learning approach that simultaneously identifies disease subtypes and their progression trajectories from cross-sectional data [5, 16]. The algorithm clusters individuals into subtypes that share common temporal patterns of disease progression, while inferring the sequential order of biomarker abnormalities within each subtype.

The analysis was implemented using the publicly available Python package *pySuStaIn* (https://github.com/ucl-pond/pySuStaIn) [16]. We utilised the z-score model, defining disease events as deviations of one, two, and three standard deviations from the healthy control mean. Model selection was based on the Bayesian Information Criterion, and participants were assigned to subtypes and stages using maximum likelihood estimation [17]. Disease progression patterns were visualised using brain rendering and matrix-based heat maps (see Supplementary Methods S4 for detailed parameters and procedures).

### Functional MRI analysis

#### Data acquisition and preprocessing

fMRI data were preprocessed using Statistical Parametric Mapping (SPM12) software (Wellcome Trust Centre for Neuroimaging, London, UK) following standard procedures [18, 19]. Preprocessing steps included slice timing correction, realignment, spatial normalisation to the Montreal Neurological Institute (MNI) template [20], and smoothing with a FWHM kernel of twice the voxel size.

Preprocessed data were further analysed using the CONN toolbox, version conn22a [21]. Signals related to the six head-motion parameters as well as white matter and cerebrospinal fluid signals were regressed out. The residual BOLD signals were linearly detrended and band-pass filtered (0.008–0.09 Hz). A functional connectivity matrix was generated for each participant using the JHU atlas as the basis for 96 ROIs [14]. Each functional connectivity matrix element represented the Fisher-transformed bivariate correlation coefficient between the BOLD time series from pairs of ROIs.

### Statistical analysis

#### Clinical and demographic comparisons

Between-group comparisons (patients vs. healthy controls, and between subtypes) were performed using Mann-Whitney U tests for continuous variables and chi-square tests for categorical variables. To ensure that identified clinical differences were not confounded by disease progression stage, ANCOVA was performed with stage as a covariate and stage-by-subtype interaction as an additional factor.

To examine associations between disease progression stage and clinical characteristics within each subtype, multiple linear regression analyses were conducted with age and sex included as covariates.

#### Between-group functional connectivity analysis

To examine group differences in functional connectivity between schizophrenia subtypes, we employed Network-Based Statistics (NBS) version 1.2 [22]. A general linear model was constructed with age, sex, handedness, scanner type, and disease progression stage included as covariates. Group comparisons were performed using *t*-tests with 5000 permutations. The significance threshold was set at *p* < 0.05, corrected for multiple comparisons using the FDR method. To ensure that identified connectivity differences were not confounded by disease progression stage, ANCOVA was performed for significant connections with stage as a covariate and stage-by-subtype interaction as an additional factor.

#### Disease stage associations analysis

To investigate associations between disease progression and both structural changes and functional connectivity patterns within each subtype, we employed a permutation-based partial correlation approach for both analyses.

For structural data, partial Spearman’s rank correlation analysis was performed between disease progression stage and regional brain volumes. Age, sex, handedness, and TIV were included as covariates in the partial correlation analysis.

For functional connectivity data, partial Spearman’s rank correlation analysis was performed between disease progression stage and all possible pairwise connections among the 96 brain regions (yielding 4560 unique connections). Age, sex, handedness, and scanner type were included as covariates in the partial correlation analysis.

For both analyses, variables were first regressed against the covariates using linear regression, and Spearman’s rank correlations were then computed using the residuals. Statistical significance was assessed using permutation testing with 20,000 permutations. For each permutation, the stage residuals were randomly shuffled while preserving the original data structure, and partial correlation coefficients were recalculated. P-values were determined as the proportion of permuted correlations exceeding the observed absolute correlation.

Multiple comparison correction was applied using two approaches. First, False Discovery Rate (FDR) correction was performed using the Benjamini-Hochberg procedure with significance defined as FDR q < 0.05. Second, Family-Wise Error Rate (FWER) control was applied using the permutation-based maximum statistic method, which accounts for the correlation structure among connectivity measures while controlling FWER at α = 0.05. All statistical analyses were performed using Python 3.10.

## Results

### Participant characteristics

The original dataset included 92 patients with schizophrenia and 234 healthy controls. We excluded 7 patients due to missing MRI data or severe motion artifacts, and 10 controls due to outlier brain volumetric measures. The final analytic sample comprised 85 patients and 224 controls (Table 1). Exclusion criteria are detailed in Supplementary Figure S5. All participants underwent MRI scanning at a single facility using two 3.0 Tesla systems. Clinical assessments including PANSS and Brief Assessment of Cognition in Schizophrenia (BACS) were available for most patients, with a subset of controls also completing BACS (Table 1).

**Table 1:** Demographic and Clinical Characteristics.

Characteristic	Healthy Controls (n = 224)	Schizophrenia Patients (n = 85)	p value
**Age (years), mean** **±** **SD**	34.5 ± 12.8	39.8 ± 10.4	< 0.001
**Sex, n (%)**			0.195
**Male**	136 (60.7%)	44 (51.8%)	
**Female**	88 (39.3%)	41 (48.2%)	
**Handedness, n (%)**			0.415
**Right-handed**	215 (96%)	79 (92.9%)	
**Left-handed**	9 (4.0%)	6 (7.1%)	
**MRI Scanner, n (%)**			< 0.001
**SIEMENS Trio**	65 (29.0%)	46 (54.1%)	
**SIEMENS TimTrio**	159 (71.0%)	39 (45.9%)	
**Duration of illness (years), mean** **±** **SD**	-	14.6 ± 9.5 (n = 84)	
**Antipsychotic treatment, n (%)**	-	83/85 (97.6%)	
**Antidepressant use, n (%)**	-	11/85 (12.9%)	
**PANSS scores, mean** **±** **SD**			
**Positive symptoms**	-	13.8 ± 5.0 (n = 81)	
**Negative symptoms**	-	15.7 ± 5.9 (n = 82)	
**General psychopathology**	-	29.6 ± 9.2 (n = 82)	
**Total score**	-	59.2 ± 17.9 (n = 81)	
**BACS scores, mean** **±** **SD**			
**Verbal Memory**	51.6 ± 9.3 (n = 150)	37.8 ± 11.5 (n = 81)	< 0.001
**Verbal Fluency**	51.4 ± 11.1 (n = 150)	40.7 ± 10.8 (n = 81)	< 0.001
**Working Memory**	22.4 ± 4.0 (n = 150)	18.5 ± 4.1 (n = 81)	< 0.001
**Motor Speed**	79.5 ± 13.9 (n = 150)	62.1 ± 17.3 (n = 81)	< 0.001
**Attention/Processing Speed**	73.1 ± 13.2 (n = 150)	52.3 ± 13.1 (n = 81)	< 0.001
**Executive Function**	18.7 ± 2.5 (n = 150)	17.5 ± 3.6 (n = 81)	< 0.001

Data are presented as mean ± standard deviation for continuous variables and number (percentage) for categorical variables. PANSS and BACS assessments are available for subsets of participants, as indicated by the sample sizes in parentheses. Statistical comparisons were performed using Mann-Whitney U tests for continuous variables and chi-square tests for categorical variables.

*SD*, standard deviation; *PANSS*, positive and negative syndrome scale; *BACS*, brief assessment of cognition in schizophrenia.

### SuStaIn-derived subtypes

**Fig. 1:**
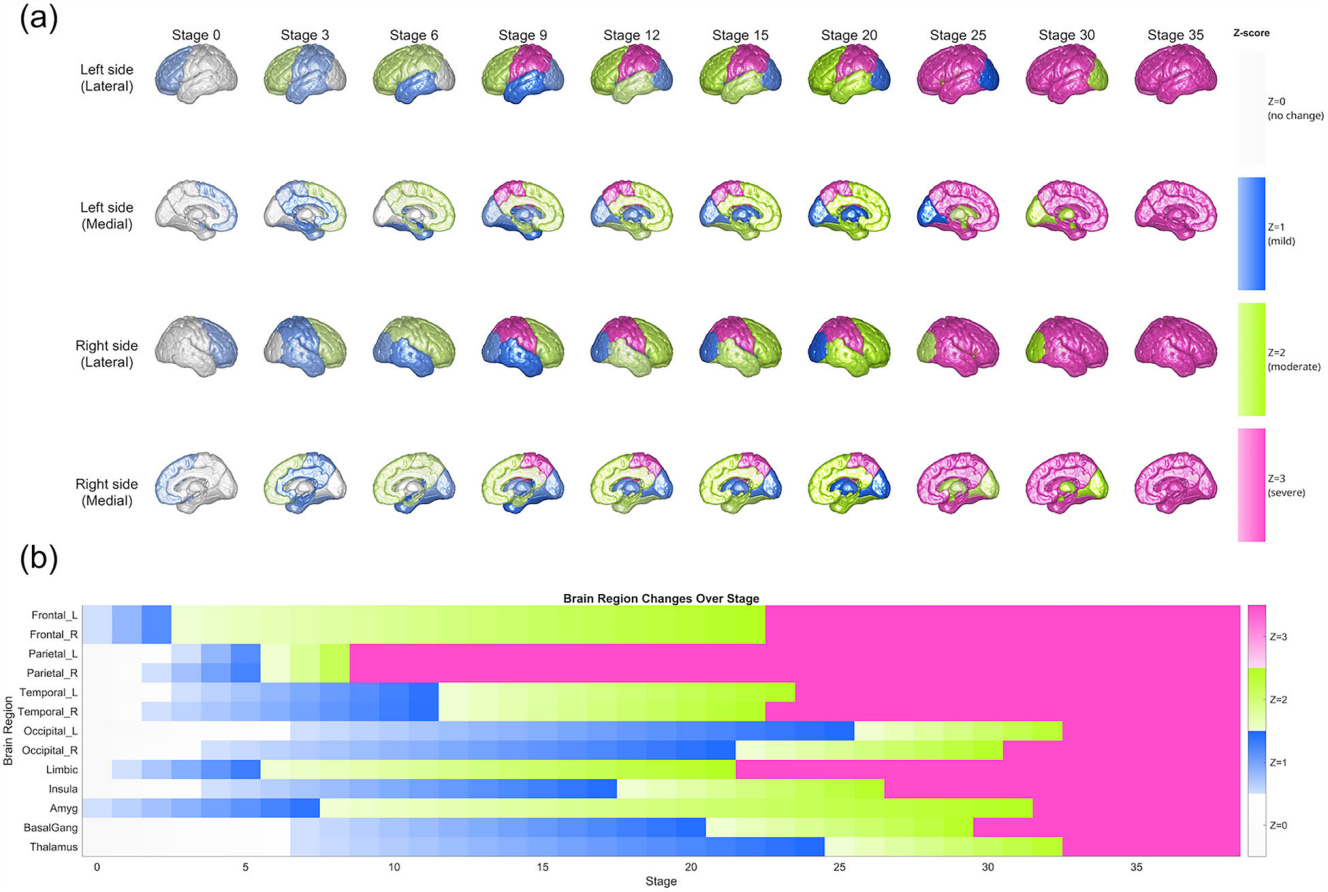
Spatiotemporal progression patterns of brain atrophy in Subtype0 of schizophrenia identified by SuStaIn algorithm. (**a**) Brain renderings and (**b**) heat maps showing regional atrophy progression across disease stages for Subtype0 (anterior-onset). Color intensity represents atrophy severity as z-scores relative to healthy controls: no change (gray/light blue), mild (z = 1), moderate (z = 2), and severe atrophy (magenta, z = 3). Subtype0 demonstrates anterior-to-posterior progression from frontal-limbic regions. L = left; R = right; Amyg = amygdala; BasalGang = basal ganglia.

Subtype0 exhibited an anterior-to-posterior progression, with early frontal lobe and amygdala atrophy (z-score = 1.0) evident from Stage0, followed by rapid parietal deterioration (Stages 3–9) and gradual temporal involvement. The occipital lobe, thalamus, and basal ganglia remained largely preserved until the later stages, with all regions reaching maximum severity by approximately Stage 33 (Fig. 1).

**Fig. 2:**
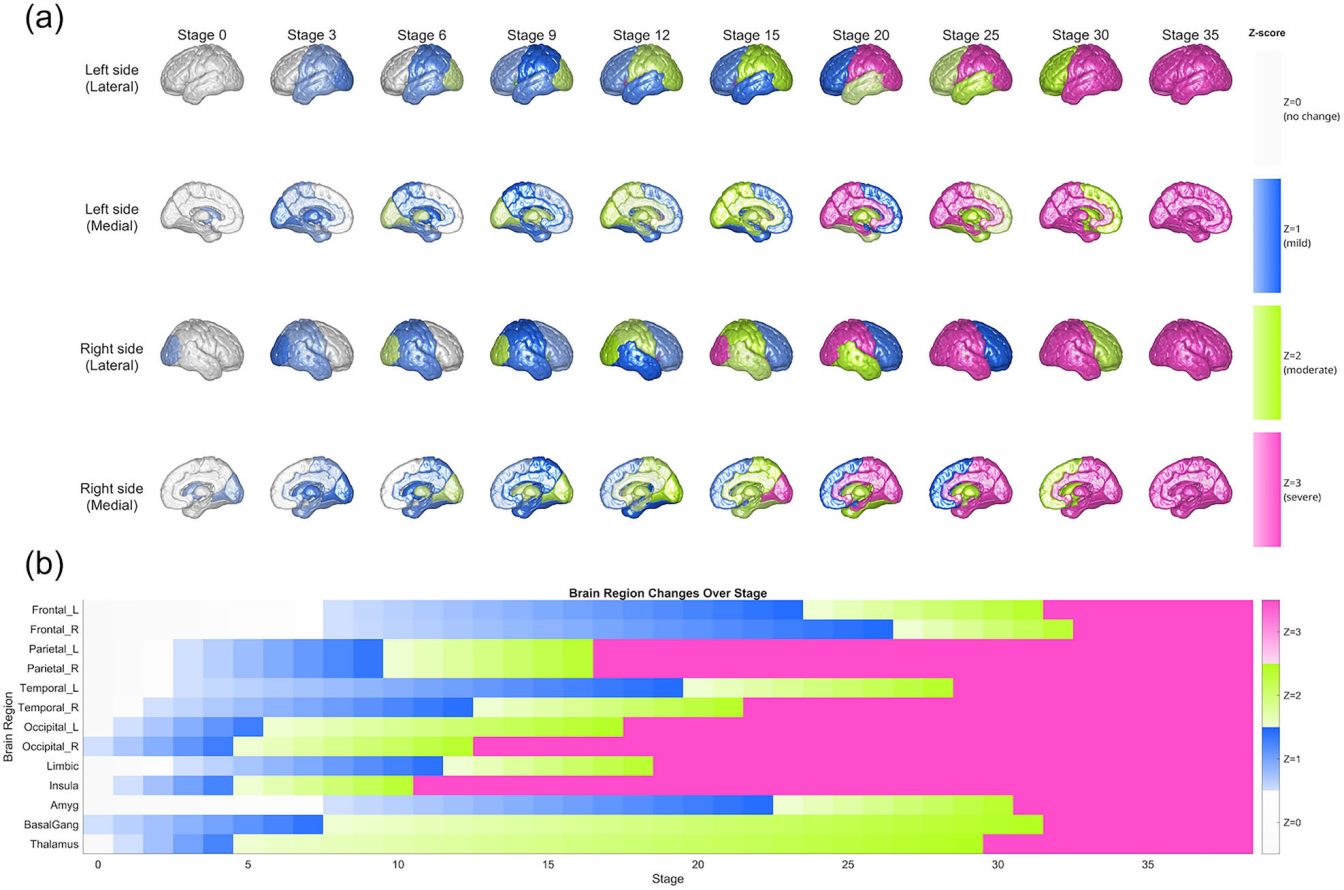
Spatiotemporal progression patterns of brain atrophy in Subtype1 of schizophrenia identified by SuStaIn algorithm. (**a**) Brain renderings and (**b**) heat maps showing regional atrophy progression across disease stages for Subtype1 (posterior-onset). Color intensity represents atrophy severity as z-scores relative to healthy controls: no change (gray/light blue), mild (z = 1), moderate (z = 2), and severe atrophy (magenta, z = 3). Subtype1 demonstrates posterior-to-anterior progression originating from thalamus, basal ganglia, and occipital cortex, followed by temporal and parietal involvement. L = left; R = right; Amyg = amygdala; BasalGang = basal ganglia.

Subtype1 showed a contrasting posterior-to-anterior progression, originating from the thalamus, basal ganglia, and right occipital lobe at Stage 0. Subsequent atrophy involved the insular and temporal cortices, followed by the parietal regions (Stages 9–16), with frontal changes occurring relatively late. Maximum severity was reached around Stage 32 (Fig. 2).

### Clinical differences between subtypes

**Fig. 3:**
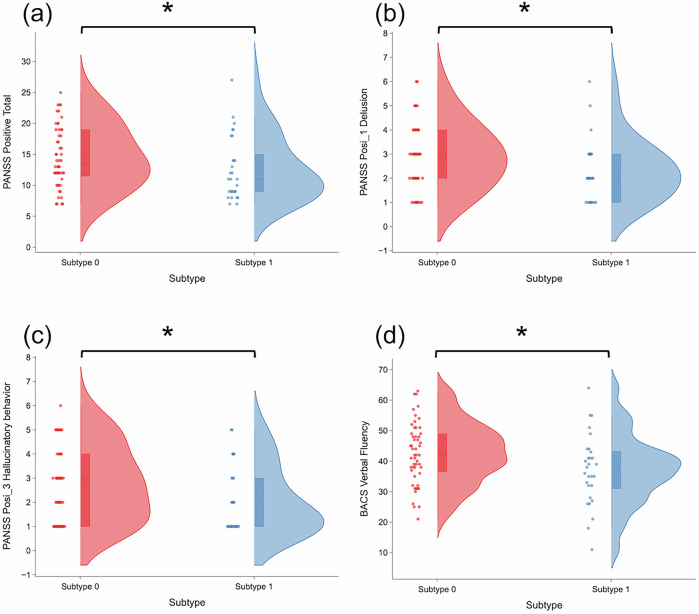
Clinical differences between structural subtypes identified by SuStaIn analysis. Violin plots comparing Subtype0 (anterior-onset, red) and Subtype1 (posterior-onset, blue). (**a**) PANSS positive symptom total scores (14.66 vs 12.55, p = 0.044). (**b**) PANSS delusions subscale scores (3.69 vs 2.27, p = 0.034). (**c**) PANSS hallucinatory behavior subscale scores (3.73 vs 2.87, p = 0.026). (**d**) BACS verbal fluency scores (43.25 vs 37.61, p = 0.042). Individual data points are overlaid on distributions. Asterisks (*) indicate statistically significant differences (p < 0.05, Mann-Whitney U tests). Abbreviations: PANSS = Positive and Negative Syndrome Scale; BACS = Brief Assessment of Cognition in Schizophrenia.

Significant differences were observed between subtypes in both psychiatric symptoms and cognitive performance (Fig. 3, Supplementary Table S6).

#### Psychiatric symptoms

Subtype0 demonstrated significantly higher positive symptoms compared to Subtype1, independent of disease progression stage (Supplementary Table S6). This included higher PANSS positive subscale scores (14.66 vs 12.55, p = 0.044), delusions (3.69 vs 2.27, p = 0.034), and hallucinatory behaviour (3.73 vs 2.87, p = 0.026) (Fig. 3a–c). No significant differences were observed in negative symptoms or general psychopathology scores.

#### Cognitive function

Subtype0 exhibited significantly better performance on the verbal fluency task compared to Subtype1 (43.25 vs 37.61, *p* = 0.042) (Fig. 3d). No significant differences were found in other cognitive domains.

ANCOVA confirmed that these subtype differences were not influenced by disease progression stage, with no significant main effects of stage (all F < 1.40, p > 0.24) or subtype-by-stage interactions (all F < 0.26, p > 0.61) across all clinical measures.

#### Stage-related clinical factors

Distinct stage-related clinical correlates were identified for each subtype. In Subtype0, hostility was most strongly associated with disease stage (β = 0.735, p = 0.009), while in Subtype1, passive/apathetic social withdrawal showed the strongest negative association (β = −1.052, p = 0.030). To assess potential medication confounding, we examined associations between disease stage and medication variables. No significant correlations were observed with antipsychotic dose, illness duration, or concomitant medication use (Supplementary Table S7).

### Structure–stage associations

Distinct patterns of association between regional brain volumes and disease progression stage were observed for the two subtypes (Supplementary Table S8).

#### Subtype0

Permutation-based partial correlation analysis identified 16 brain regions showing significant negative associations with disease progression stage (FDR q < 0.05). The strongest correlations were observed in the right precuneus (ρ = −0.48), bilateral dorsal anterior cingulate cortex (right: ρ = −0.45; left: ρ = −0.43), bilateral rectus gyrus (right: ρ = −0.42; left: ρ = −0.41), and left supramarginal gyrus (ρ = −0.43). Additional significant associations included bilateral middle frontal gyrus, posterior cingulate cortex, angular gyrus, parahippocampal gyrus, superior parietal gyrus, and inferior frontal gyrus orbitalis (ρ = −0.35 to −0.38, Supplementary Table S8). Scatter plots illustrating these correlations are presented in Supplementary Figure S9.

#### Subtype1

For Subtype1, five brain regions showed associations with disease progression stage based on uncorrected p-values, though none survived FDR correction. The strongest correlation was observed in the right cuneus (ρ = −0.54), followed by the right lingual gyrus (ρ = −0.47), left posterior basal forebrain (ρ = 0.44), right middle occipital gyrus (ρ = −0.39), and right dorsal anterior cingulate cortex (ρ = −0.37) (Supplementary Table S8). Scatter plots illustrating these correlations are presented in Supplementary Figure S10.

### Functional connectivity findings

#### Between-subtype differences

NBS analysis identified a single connection that significantly differed between Subtype0 and Subtype1 (FDR-corrected p < 0.05). This was the right cuneus-right thalamus connection, with Subtype1 showing stronger connectivity. ANCOVA confirmed that this difference was not confounded by disease progression stage (stage effect F = 0.13, p = 0.718; stage-by-subtype interaction F = 0.24, p = 0.625) or scanner type (F = 0.41, p = 0.523).

#### Stage-related patterns

To investigate associations between disease progression and functional connectivity, we analysed all 4560 pairwise connections among the 96 brain regions using permutation-based partial correlation analysis. Age, sex, handedness, and scanner type were included as covariates in all models, and no significant scanner effects were identified for the reported connections.

#### Subtype0

**Fig. 4:**
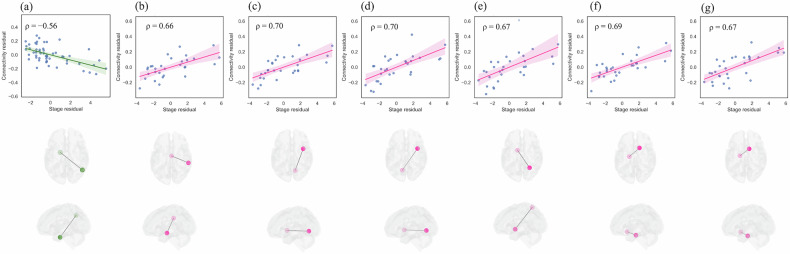
Contrasting functional connectivity changes across disease progression stages in schizophrenia subtypes. Stage-connectivity correlations after adjusting for age, sex, handedness, and scanner type. Regression lines with 95% confidence intervals (shaded areas) are shown. (**a**) Subtype0: Progressive hypoconnectivity in right angular gyrus—left entorhinal cortex (FWER-corrected p < 0.05). (**b**-**g**) Subtype1: Progressive hyperconnectivity in six connections (all p < 0.0001, uncorrected): (**b**) right supramarginal—right mammillary; (**c**) right lingual—right insula; (**d**) left lingual—right insula; (**e**) right superior parietal—left posterior basal forebrain; (**f**) left parahippocampal—right anterior basal forebrain; (**g**) left parahippocampal—right nucleus accumbens.

In Subtype0, no connections survived FDR correction for multiple comparisons. However, one connection survived FWER correction using the permutation-based maximum statistic method. The right angular gyrus-left entorhinal cortex connection showed a strong negative correlation with disease stage (ρ = −0.56, FWER-corrected p < 0.05, uncorrected p < 0.0001), uninfluenced by scanner effects. Figure 4a presents a scatter plot demonstrating this correlation. A complete list of connections with uncorrected p < 0.001 is provided in Supplementary Table S11.

#### Subtype1

In Subtype1, no connections survived FDR correction for multiple comparisons. However, six connections demonstrated exceptionally strong correlations (ρ = 0.66–0.70, all p < 0.0001, FDR q = 0.076). Given that these represent approximately 0.13% of the 4,560 tested connections and the rarity of such correlation coefficients in functional connectivity research, these findings likely represent biologically meaningful patterns. These included bilateral lingual gyrus-right insula connections (ρ = 0.70), left parahippocampal gyrus-right anterior basal forebrain (ρ = 0.69), right superior parietal gyrus-left posterior basal forebrain (ρ = 0.67), left parahippocampal gyrus-right nucleus accumbens (ρ = 0.67), and right supramarginal gyrus-right mammillary body (ρ = 0.66). None were influenced by scanner effects. Figure 4b–g presents scatter plots demonstrating these correlations. A complete list of connections with uncorrected p < 0.001 is provided in Supplementary Table S12.

## Discussion

We integrated SuStaIn-based structural subtype classification with resting-state fMRI analysis in schizophrenia for the first time, identifying two distinct subtypes. Subtype0 (anterior-onset) showed progression from frontal-limbic regions posteriorly, while Subtype1 (posterior-onset) progressed from subcortical-occipital regions anteriorly. These subtypes demonstrated distinct clinical profiles, with Subtype0 exhibiting more pronounced positive symptoms and Subtype1 showing increased social withdrawal.

Crucially, we observed strong correlations between disease progression stage and functional connectivity. Subtype0 exhibited progressive hypoconnectivity in angular gyrus-entorhinal networks (ρ = −0.56), while Subtype1 displayed hyperconnectivity in lingual gyrus-insula networks (ρ = 0.70). These opposing patterns may explain conflicting functional connectivity findings in schizophrenia, as previous studies likely captured different subtypes at various disease stages. Our findings suggest structure–function relationships follow subtype-specific trajectories, potentially informing targeted therapeutic strategies.

### Clinical significance of structural subtype classification

The two structural subtypes identified by SuStaIn demonstrated distinct clinical characteristics, underscoring schizophrenia’s pathological heterogeneity. These subtypes aligned with progression patterns reported by Jiang et al. [6]. Our Subtype0 (anterior-onset) corresponds to their early cortical-predominant loss subtype, while Subtype1 (posterior-onset) corresponds to their early subcortical-predominant loss subtype.

#### Subtype0: Anterior-onset type

Subtype0 followed an anterior-to-posterior trajectory originating in frontal-limbic regions, extending Jiang et al.‘s [6] early cortical-predominant loss subtype with early amygdala involvement.

Permutation-based partial correlation analysis with FDR correction identified 16 brain regions showing significant negative associations between disease progression stage and regional volumes. These findings revealed consistent patterns of atrophy across multiple brain networks. The affected regions included core default mode network (DMN) nodes (bilateral precuneus, posterior cingulate cortex, angular gyrus, and rectus gyrus), frontoparietal network (FPN) components (middle frontal gyrus, superior frontal pole, superior parietal gyrus), and bilateral dorsal anterior cingulate cortex representing the salience network core.

FPN regions are involved in selective attention, reality testing, and behavioral control.

Moreover, bilateral dorsal anterior cingulate cortex, representing the salience network core, showed strong negative correlations with disease stage [23]. The concurrent involvement of DMN, salience network, and FPN cores aligns with reports linking their dysfunction to positive symptoms [24–29]. Given that Subtype0 exhibited atrophy across these networks, structural breakdown of major networks may underlie positive symptoms including delusions and hallucinations.

Notably, we observed a significant stage-related increase in hostility (β = 0.735, p = 0.009), likely reflecting progressive impairment of cognitive control as frontal atrophy advances. This is consistent with prior research linking frontal cortical degeneration—particularly in the anterior cingulate cortex—to increased aggressive behaviour and dysregulated responses [30–32].

#### Subtype1: Posterior-onset type

The posterior-to-anterior progression pattern observed in Subtype1 corresponds to Jiang et al.‘s [6] “early subcortical-predominant loss subtype,” with visual integration system dysfunction potentially serving as a critical initiating event. However, none of the identified regions survived FDR correction. The observed trends appear consistent with sensory processing pathway involvement.

The negative correlations in visual processing regions (right cuneus (ρ = −0.54), right lingual gyrus (ρ = −0.47), right middle occipital gyrus (ρ = −0.39)) are consistent with impairment of visual processing pathways. The positive correlation with the left posterior basal forebrain (ρ = 0.44) could indicate compensatory mechanisms [33–37]. The concurrent negative correlation with the right dorsal anterior cingulate cortex appears to reflect dysfunction in attention allocation and sensory filtering mechanisms.

The observed posterior-to-anterior progression pattern may relate to the negative association between passive/apathetic social withdrawal (PANSS N4) and disease stage (β = −1.052, p = 0.030). This pattern is consistent with early-stage sensory processing difficulties contributing to avoidance behaviors [38, 39].

This progression pattern suggests cognitive function may initially be preserved through basal forebrain circuits, with depressive or negative symptoms predominating. As pathology extends to frontal control networks, the clinical phenotype could shift toward more complex psychotic symptoms with increased treatment resistance.

These stage-dependent dynamics may explain conflicting findings regarding striatal volume and PANSS N4 associations in previous cross-sectional studies [40–43], potentially representing cross-sectional snapshots of different stages within a shared pathological trajectory.

### Medication effects and disease progression

An important consideration is the potential confounding effect of antipsychotic medication on brain structure. While longitudinal studies have reported associations between antipsychotic exposure and volume changes, a systematic review of randomized controlled trials found no significant difference between haloperidol and second-generation antipsychotics, and suggested possible neuroprotective effects [44]. Notably, placebo-controlled trials demonstrated volume decrease in untreated patients [45]. Several observations suggest our findings primarily reflect disease pathology. Disease stage showed no correlation with antipsychotic dose or illness duration, and concomitant medication use showed no association with disease stage. Nevertheless, the absence of medication duration data prevents definitive conclusions.

### Pathological specificity of functional connectivity patterns and integrated understanding of previous studies

#### Between-subtype connectivity differences

NBS analysis revealed a single statistically significant difference in functional connectivity between subtypes: the right cuneus-right thalamus connection, with Subtype1 showing stronger connectivity. ANCOVA confirmed this difference was independent of disease progression stage, indicating this connectivity pattern represents a fundamental distinguishing feature between subtypes rather than a stage-related phenomenon.

This stage-independent connectivity difference suggests cuneus-thalamus coupling may serve as a critical biomarker for subtype classification, providing a functional signature that distinguishes the two pathophysiological trajectories. This suggests visual-thalamic networks may play a key role in subtype differentiation.

#### Subtype0: Pathological hypoconnectivity patterns

In Subtype0, functional connectivity analysis revealed a single connection that survived stringent statistical correction. The right angular gyrus—left entorhinal cortex connection showed a strong negative correlation with disease progression stage (ρ = −0.56, FWER-corrected p < 0.05).

The angular gyrus serves as a posterior default mode network hub supporting self-referential processing, while the entorhinal cortex acts as the primary gateway for episodic memory encoding. Progressive weakening of connectivity between these regions suggests functional decoupling between self-referential processing and memory contextualization, aligning with previous reports of cortico-hippocampal network dysfunction in schizophrenia [46]. This functional decline likely represents structural deterioration affecting core DMN nodes, salience network components, and FPN regions in Subtype0. The disrupted integration between angular gyrus and entorhinal cortex may underlie patients’ difficulty distinguishing internally generated thoughts from external reality, providing a mechanistic explanation for the stage-related increase in hostility (β = 0.735, p = 0.009) and the positive symptom-predominant profile characteristic of Subtype0.

#### Subtype1: Pathological hyperconnectivity patterns

Functional connectivity analysis of Subtype1 revealed hyperconnectivity patterns contrasting with Subtype0. Six connections demonstrated strong associations with disease progression (ρ = 0.66–0.70, all p < 0.0001, FDR q = 0.076), including bilateral lingual gyrus-right insula connections (ρ = 0.70), parahippocampal gyrus-anterior basal forebrain (ρ = 0.69), superior parietal gyrus-posterior basal forebrain (ρ = 0.67), parahippocampal gyrus-nucleus accumbens (ρ = 0.67), and supramarginal gyrus-mammillary body (ρ = 0.66). While these connections did not survive FDR correction, the exceptionally large effect sizes rarely observed in functional connectivity research provide compelling evidence for these associations. None of these findings were influenced by scanner effects, and all remained significant when controlling for disease progression stage as a covariate. The enhanced visual-salience network coupling, particularly between lingual gyrus and right insula, may represent adaptive responses to early structural deterioration in posterior regions. The basal forebrain cholinergic system’s enhanced coupling with cortical regions suggests heightened neural activity as thalamic and subcortical structures deteriorate. Enhanced parahippocampal-nucleus accumbens connectivity aligns with previous reports of aberrant hyperconnectivity in schizophrenia [47], suggesting compensatory mechanisms within memory-reward circuits during early disease stages.

These connectivity patterns align with Subtype1’s distinctive clinical profile, particularly the negative association between passive/apathetic social withdrawal and disease stage (β = −1.052, p = 0.030). Enhanced connectivity may reflect altered network dynamics in response to early structural changes. As disease progresses, the clinical phenotype may shift toward more complex symptom presentations.

#### Therapeutic implications

The contrasting connectivity patterns—progressive disconnection in Subtype0 versus hyperconnectivity in Subtype1—indicate dynamic disease phases, suggesting therapeutic intervention windows where targeted treatments could alter trajectories. Strong stage-connectivity correlations (up to |ρ | = 0.70) suggest functional connectivity metrics could serve as treatment response biomarkers.

#### Limitations

This study has several limitations. First, due to its cross-sectional design, the disease progression stages estimated by SuStaIn do not directly capture longitudinal changes, requiring validation in future longitudinal studies. Second, the scarcity of medication-naive patients and lack of medication duration data limit our ability to fully exclude long-term cumulative effects of antipsychotic treatment on brain structure. While disease stage showed no correlation with current medication dose or concomitant medication use, detailed medication history would be necessary to definitively separate medication effects from disease progression. Third, clinical assessments were missing for some participants, limiting complete clinical characterisation of the cohort.

We identified two biologically distinct subtypes with unique spatiotemporal progression patterns. Subtype0 (anterior-onset) exhibited progressive connectivity decline (maximum ρ = −0.56), while Subtype1 (posterior-onset) showed progressive connectivity enhancement (maximum ρ = 0.70). These contrasting patterns may explain previously contradictory functional connectivity findings in schizophrenia. Our findings suggest that structure-function relationships do not necessarily occur in parallel and follow subtype-specific trajectories, providing foundational evidence for developing targeted therapeutic strategies.

## Supplementary information

Supplementary Information

## Data Availability

Preprocessing scripts and the pySuStaIn model are available at https://github.com/ucl-pond/pySuStaIn. Additional analysis code will be made available upon reasonable request. The original data are available from the DecNef Project Brain Data Repository (https://bicr-resource.atr.jp/srpbsopen/) upon reasonable request and with appropriate approval from the participating institutions.

## References

[1] van Os J, Kapur S. Schizophrenia. Lancet. 2009;374:635–45. 19700006 10.1016/S0140-6736(09)60995-8

[2] Mattila T, Koeter M, Wohlfarth T, Storosum J, van den Brink W, de Haan L, et al. Impact of DSM-5 Changes on the diagnosis and acute treatment of schizophrenia. Schizophr Bull. 2015;41:637–43. 25528758 10.1093/schbul/sbu172 PMC4393695

[3] Braff DL, Ryan J, Rissling AJ, Carpenter WT. Lack of use in the literature from the last 20 years supports dropping traditional schizophrenia subtypes from DSM-5 and ICD-11. Schizophr Bull. 2013;39:751–3. 23674819 10.1093/schbul/sbt068 PMC3686462

[4] Clementz BA, Sweeney JA, Hamm JP, Ivleva EI, Ethridge LE, Pearlson GD, et al. Identification of distinct psychosis biotypes using brain-based biomarkers. Am J Psychiatry. 2016;173:373–84. 26651391 10.1176/appi.ajp.2015.14091200 PMC5314432

[5] Young AL, Marinescu RV, Oxtoby NP, Bocchetta M, Yong K, Firth NC, et al. Uncovering the heterogeneity and temporal complexity of neurodegenerative diseases with Subtype and Stage Inference. Nat Commun. 2018;9:4273. 30323170 10.1038/s41467-018-05892-0 PMC6189176

[6] Jiang Y, Luo C, Wang J, Palaniyappan L, Chang X, Xiang S, et al. Neurostructural subgroup in 4291 individuals with schizophrenia identified using the subtype and stage inference algorithm. Nat Commun. 2024;15:5996. 39013848 10.1038/s41467-024-50267-3 PMC11252381

[7] Sone D, Young A, Shinagawa S, Tsugawa S, Iwata Y, Tarumi R, et al. Disease progression patterns of brain morphology in schizophrenia: more progressed stages in treatment resistance. Schizophr Bull. 2023;50:393–402. 10.1093/schbul/sbad164 PMC10919766 38007605

[8] Zhao W, Zhang X, Zhou X, Song X, Zhang Z, Xu L, et al. Depression mediates the association between insula-frontal functional connectivity and social interaction anxiety. Hum Brain Mapp. 2022;43:4266–73. 35596617 10.1002/hbm.25952 PMC9435016

[9] Whitfield-Gabrieli S, Thermenos HW, Milanovic S, Tsuang MT, Faraone SV, McCarley RW, et al. Hyperactivity and hyperconnectivity of the default network in schizophrenia and in first-degree relatives of persons with schizophrenia. Proc Natl Acad Sci USA. 2009;106:1279–84. 19164577 10.1073/pnas.0809141106 PMC2633557

[10] Dabiri M, Dehghani Firouzabadi F, Yang K, Barker PB, Lee RR, Yousem DM Neuroimaging in schizophrenia: A review article. Front Neurosci 2022; **16**. 10.3389/fnins.2022.1042814. 10.3389/fnins.2022.1042814 PMC9706110 36458043

[11] Ruiz-Torras S, Gudayol-Ferré E, Fernández-Vazquez O, Cañete-Massé C, Peró-Cebollero M, Guàrdia-Olmos J. Hypoconnectivity networks in schizophrenia patients: A voxel-wise meta-analysis of Rs-fMRI. Int J Clin Health Psychol. 2023;23:100395 10.1016/j.ijchp.2023.100395. 37533450 10.1016/j.ijchp.2023.100395 PMC10392089

[12] Chen P, Ye E, Jin X, Zhu Y, Wang L. Association between thalamocortical functional connectivity abnormalities and cognitive deficits in schizophrenia. Sci Rep. 2019;9:2952. 30814558 10.1038/s41598-019-39367-z PMC6393449

[13] Sliz D, Hayley S. Major depressive disorder and alterations in insular cortical activity: A review of current functional magnetic imaging research. Front Hum Neurosci. 2012;6:323. 23227005 10.3389/fnhum.2012.00323 PMC3512092

[14] Oishi K, Faria A, Jiang H, Li X, Akhter K, Zhang J, et al. Atlas-based whole brain white matter analysis using large deformation diffeomorphic metric mapping: Application to normal elderly and Alzheimer’s disease participants. Neuroimage. 2009;46:486–99. 19385016 10.1016/j.neuroimage.2009.01.002 PMC2885858

[15] Nishimaki K, Onda K, Ikuta K, Chotiyanonta J, Uchida Y, Mori S, et al. OpenMAP-T1: A rapid deep-learning approach to parcellate 280 anatomical regions to cover the whole brain. Hum Brain Mapp. 2024;45:e70063. 39523990 10.1002/hbm.70063 PMC11551626

[16] Aksman LM, Wijeratne PA, Oxtoby NP, Eshaghi A, Shand C, Altmann A, et al. pySuStaIn: a Python implementation of the Subtype and Stage Inference algorithm. SoftwareX. 2021;16:100811. 34926780 10.1016/j.softx.2021.100811 PMC8682799

[17] Burnham KP, Anderson DR Model selection and multi-model inference: a practical information-theoretic approach. New York London: Springer; 2011. New ed.

[18] Biswal BB, Uddin LQ. The history and future of resting-state functional magnetic resonance imaging. Nature. 2025;641:1121–31. 40437164 10.1038/s41586-025-08953-9 PMC12628496

[19] Di X, Biswal BB. A functional MRI pre-processing and quality control protocol based on statistical parametric mapping (SPM) and MATLAB. Front Neuroimaging. 2023;1:1070151. 37555150 10.3389/fnimg.2022.1070151 PMC10406300

[20] Fonov V, Evans AC, Botteron K, Almli CR, McKinstry RC, Collins DL. Unbiased average age-appropriate atlases for pediatric studies. Neuroimage. 2011;54:313–27. 20656036 10.1016/j.neuroimage.2010.07.033 PMC2962759

[21] Liu TT, Falahpour M Vigilance effects in resting-state fMRI. *Front Neurosci* 2020; **14**. 10.3389/fnins.2020.00321. 10.3389/fnins.2020.00321 PMC7190789 32390792

[22] Zalesky A, Fornito A, Bullmore ET. Network-based statistic: Identifying differences in brain networks. Neuroimage. 2010;53:1197–207. 20600983 10.1016/j.neuroimage.2010.06.041

[23] Schimmelpfennig J, Topczewski J, Zajkowski W, Jankowiak-Siuda K. The role of the salience network in cognitive and affective deficits. Front Hum Neurosci. 2023;17:1133367 10.3389/fnhum.2023.1133367. 37020493 10.3389/fnhum.2023.1133367 PMC10067884

[24] Hare SM, Ford JM, Mathalon DH, Damaraju E, Bustillo J, Belger A, et al. Salience–Default mode functional network connectivity linked to positive and negative symptoms of schizophrenia. Schizophr Bull. 2019;45:892–901. 30169884 10.1093/schbul/sby112 PMC6581131

[25] Brakowski J, Manoliu A, Homan P, Bosch OG, Herdener M, Seifritz E, et al. Aberrant striatal coupling with default mode and central executive network relates to self-reported avolition and anhedonia in schizophrenia. J Psychiatr Res. 2022;145:263–75. 33187692 10.1016/j.jpsychires.2020.10.047

[26] Zhu T, Wang Z, Wu W, Ling Y, Wang Z, Zhou C et al. Altered brain functional networks in schizophrenia with persistent negative symptoms: an activation likelihood estimation meta-analysis. Front Hum Neurosci 2023;17. 10.3389/fnhum.2023.1204632. 10.3389/fnhum.2023.1204632 PMC10637389 37954938

[27] Jáni M, Kikinis Z, Lošák J, Pasternak O, Szczepankiewicz F, Heller C, et al. Emotional awareness in schizophrenia is associated with gray matter volume of right precuneus. Front Psychiatry. 2021;12:601742. 33868042 10.3389/fpsyt.2021.601742 PMC8046932

[28] Margulies DS, Vincent JL, Kelly C, Lohmann G, Uddin LQ, Biswal BB, et al. Precuneus shares intrinsic functional architecture in humans and monkeys. Proc Natl Acad Sci USA. 2009;106:20069–74. 19903877 10.1073/pnas.0905314106 PMC2775700

[29] Dong D, Wang Y, Chang X, Luo C, Yao D. Dysfunction of large-scale brain networks in schizophrenia: A meta-analysis of resting-state functional connectivity. Schizophr Bull. 2018;44:168–81. 28338943 10.1093/schbul/sbx034 PMC5767956

[30] Liu F, Shao Y, Li X, Liu L, Zhao R, Xie B, et al. Volumetric abnormalities in violent schizophrenia patients on the general psychiatric ward. Front Psychiatry. 2020;11:788. 33117201 10.3389/fpsyt.2020.00788 PMC7493665

[31] Lamsma J, Raine A, Kia SM, Cahn W, Arold D, Banaj N, et al. Structural brain abnormalities and aggression in schizophrenia: mega-analysis of data from 2095 patients and 2861 healthy controls via the ENIGMA consortium. Mol Psychiatry. 2025. 10.1038/s41380-025-03365-7. 10.1038/s41380-025-03365-7 41339704

[32] Dehaene S, Artiges E, Naccache L, Martelli C, Viard A, Schürhoff F, et al. Conscious and subliminal conflicts in normal subjects and patients with schizophrenia: The role of the anterior cingulate. Proc Natl Acad Sci. 2003;100:13722–7. 14597698 10.1073/pnas.2235214100 PMC263880

[33] Sabaroedin K, Tiego J, Fornito A. Circuit-based approaches to understanding corticostriatothalamic dysfunction across the psychosis continuum. Biol Psychiatry. 2023;93:113–24. 36253195 10.1016/j.biopsych.2022.07.017

[34] Tarcijonas G, Foran W, Haas GL, Luna B, Sarpal DK. Intrinsic connectivity of the globus pallidus: An uncharted marker of functional prognosis in people with first-episode schizophrenia. Schizophr Bull. 2020;46:184–92. 31150557 10.1093/schbul/sbz034 PMC6942165

[35] Ananth MR, Rajebhosale P, Kim R, Talmage DA, Role LW. Basal forebrain cholinergic signalling: development, connectivity and roles in cognition. Nat Rev Neurosci. 2023;24:233–51. 36823458 10.1038/s41583-023-00677-x PMC10439770

[36] Avram M, Brandl F, Bäuml J, Sorg C. Cortico-thalamic hypo- and hyperconnectivity extend consistently to basal ganglia in schizophrenia. Neuropsychopharmacology. 2018;43:2239–48. 29899404 10.1038/s41386-018-0059-z PMC6135808

[37] Avram M, Grothe MJ, Meinhold L, Leucht C, Leucht S, Borgwardt S, et al. Lower cholinergic basal forebrain volumes link with cognitive difficulties in schizophrenia. Neuropsychopharmacol. 2021;46:2320–9. 10.1038/s41386-021-01070-x PMC8580980 34188186

[38] Adámek P, Langová V, Horáček J. Early-stage visual perception impairment in schizophrenia, bottom-up and back again. Schizophr. 2022;8:1–12. 10.1038/s41537-022-00237-9 PMC8938488 35314712

[39] Sharkey RJ, Bacon C, Peterson Z, Rootes-Murdy K, Salvador R, Pomarol-Clotet E, et al. Differences in the neural correlates of schizophrenia with positive and negative formal thought disorder in patients with schizophrenia in the ENIGMA dataset. Mol Psychiatry. 2024;29:3086–96. 38671214 10.1038/s41380-024-02563-z PMC11449795

[40] Burrer A, Caravaggio F, Manoliu A, Plitman E, Gütter K, Habermeyer B, et al. Apathy is not associated with reduced ventral striatal volume in patients with schizophrenia. Schizophr Res. 2020;223:279–88. 32928618 10.1016/j.schres.2020.08.018

[41] Kirschner M, Schmidt A, Hodzic-Santor B, Burrer A, Manoliu A, Zeighami Y, et al. Orbitofrontal-Striatal structural alterations linked to negative symptoms at different stages of the schizophrenia spectrum. Schizophr Bull. 2020;47:849–63. 10.1093/schbul/sbaa169 PMC8084448 33257954

[42] Roth RM, Garlinghouse MA, Flashman LA, Koven NS, Pendergrass JC, Ford JC, et al. Apathy is associated with ventral striatum volume in schizophrenia spectrum disorder. J Neuropsychiatry Clin Neurosci. 2016;28:191–4. 26900738 10.1176/appi.neuropsych.15100241 PMC5023440

[43] Caravaggio F, Fervaha G, Iwata Y, Plitman E, Chung JK, Nakajima S, et al. Amotivation is associated with smaller ventral striatum volumes in elderly patients with schizophrenia. Int J Geriatr Psychiatry. 2018;33:523–30. 29110353 10.1002/gps.4818 PMC5807115

[44] Fountoulakis KN, Stahl SM. The effect of first- and second-generation antipsychotics on brain morphology in schizophrenia: A systematic review of longitudinal magnetic resonance studies with a randomized allocation to treatment arms. J Psychopharmacol. 2022;36:428–38. 35395911 10.1177/02698811221087645

[45] Chopra S, Fornito A, Francey SM, O’Donoghue B, Cropley V, Nelson B, et al. Differentiating the effect of antipsychotic medication and illness on brain volume reductions in first-episode psychosis: A Longitudinal, Randomised, Triple-blind, Placebo-controlled MRI Study. Neuropsychopharmacology. 2021;46:1494–501. 33637835 10.1038/s41386-021-00980-0 PMC8209146

[46] Xue K, Chen J, Wei Y, Chen Y, Han S, Wang C, et al. Impaired large-scale cortico–hippocampal network connectivity, including the anterior temporal and posterior medial systems, and its associations with cognition in patients with first-episode schizophrenia. Front Neurosci. 2023;17:1167942. 37342466 10.3389/fnins.2023.1167942 PMC10277613

[47] Zhou C, Xue C, Chen J, Amdanee N, Tang X, Zhang H, et al. Altered functional connectivity of the nucleus accumbens network between deficit and non-deficit schizophrenia. Front Psychiatry. 2021;12:704631. 34658949 10.3389/fpsyt.2021.704631 PMC8514672

