# Supplementary material for: Data-driven schizophrenia subtyping via brain atrophy trajectories and functional connectivity
Source: Transl Psychiatry. 2026 Mar 19;16:229. doi: 10.1038/s41398-026-03968-w (PMC13043883; doi:10.1038/s41398-026-03968-w)
Supplement: Supplementary file 1 — Supplementary Information [file 41398_2026_3968_MOESM1_ESM.pdf]

Data-driven schizophrenia subtyping via brain atrophy trajectories and functional connectivity

Supplementary Materials

SUPPLEMENTARY TABLE S1. MRI ACQUISITION PARAMETERS FOR STRUCTURAL T1-WEIGHTED AND RESTING-STATE FMRI SEQUENCES..2

SUPPLEMENTARY METHODS S2. OPENMAP-T1 IMPLEMENTATION DETAILS.................3

SUPPLEMENTARY TABLE S3. BRAIN REGION DEFINITIONS...4

SUPPLEMENTARY METHODS S4. SUSTAIN ANALYSIS TECHNICAL DETAILS...................6

SUPPLEMENTARY FIGURE S5: FLOW OF PARTICIPANT SELECTION. 9

SUPPLEMENTARY TABLE S6. DEMOGRAPHIC AND CLINICAL CHARACTERISTICS BY SUBTYPE...10

SUPPLEMENTARY TABLE S7. ASSOCIATIONS BETWEEN DISEASE STAGE AND MEDICATION VARIABLES...11

SUPPLEMENTARY TABLE S8..12

SUPPLEMENTARY FIGURE S9: PARTIAL CORRELATIONS BETWEEN DISEASE PROGRESSION STAGE AND REGIONAL BRAIN VOLUMES IN SUBTYPE0 AFTER COVARIATE ADJUSTMENT 13

SUPPLEMENTARY FIGURE S10: PARTIAL CORRELATIONS BETWEEN DISEASE PROGRESSION STAGE AND REGIONAL BRAIN VOLUMES IN SUBTYPE1 AFTER COVARIATE ADJUSTMENT 14

SUPPLEMENTARY TABLE S11: FUNCTIONAL CONNECTIVITY CORRELATIONS WITH DISEASE PROGRESSION STAGE IN SUBTYPE0 15

SUPPLEMENTARY TABLE S12: FUNCTIONAL CONNECTIVITY CORRELATIONS WITH DISEASE PROGRESSION STAGE IN SUBTYPE1 16

REFERENCE 17

Supplementary Table S1. MRI acquisition parameters for structural T1-weighted and resting-state fMRI sequences

Modality	Parameter	Tim Trio	Trio
**Structural T1**	Sequence	MPRAGE	MPRAGE
	TR (ms)	2000	2000
	TE (ms)	3.4	4.38
	Flip angle (degree)	8	8
	TI (ms)	990	990
	FOV	225×240	225×240
	Matrix	240×256	240×256
	Slice thickness (mm)	1	1
**Resting-state fMRI**	TR (ms)	2500	2000
	TE (ms)	30	30
	Flip angle (degree)	80	90
	FOV	212×212	256×192
	Matrix	64×64	64×48
	Slice thickness (mm)	3.2	4
	Number of slices	40	30
	Number of volumes	240	182
	Scan duration (min)	10	6

Acquisition parameters for structural T1-weighted MPRAGE and resting-state functional MRI sequences across Tim Trio and Trio scanner systems. TR = repetition time; TE = echo time; TI = inversion time; FOV = field of view.

Supplementary Methods S2. OpenMAP-T1 Implementation Details

OpenMAP-T1 (Open resource for Multiple Anatomical structure Parcellation for T1-weighted brain MRI) is a deep learning-based method designed for rapid and accurate whole-brain parcellation into 280 anatomical regions covering both gray and white matter structures[1].

The OpenMAP-T1 preprocessing pipeline included N4 bias field correction to remove intensity inhomogeneity, reorientation to standard orientation (RAS format), interpolation to $1 \times 1 \times 1$ mm^3 isotropic voxels, and intensity normalization to a range between -1 and 1 [1–3].

The parcellation process integrates several convolutional neural network models across six phases: preprocessing, cropping (using CNet, a 2D U-Net architecture), skull-stripping (using SSNet, a 2D U-Net architecture), parcellation (using PNet, a 2.5D U-Net architecture), hemisphere segmentation (using HNet, a 2D U-Net architecture), and final merging of all regions[14]. The parcellation was based on the Johns Hopkins University (JHU)-MNI atlas[5].

Supplementary Table S3. Brain Region Definitions

Macro Region	Constituent ROIs
Frontal	Superior Frontal Gyrus<br>Superior Frontal Gyrus — Prefrontal Cortex<br>Superior Frontal Gyrus — Pole<br>Middle Frontal Gyrus<br>Middle Frontal Gyrus — Dorsolateral Prefrontal Cortex<br>Inferior Frontal Gyrus — Pars Opercularis<br>Inferior Frontal Gyrus — Pars Orbitalis<br>Inferior Frontal Gyrus — Pars Triangularis<br>Lateral Fronto-Orbital Gyrus<br>Middle Fronto-Orbital Gyrus<br>Rectus Gyrus
Parietal	Postcentral Gyrus<br>Precentral Gyrus<br>Superior Parietal Gyrus<br>Supramarginal Gyrus<br>Angular Gyrus<br>Precuneus
Temporal	Superior Temporal Gyrus<br>Superior Temporal Gyrus — Pole<br>Middle Temporal Gyrus<br>Middle Temporal Gyrus — Pole<br>Inferior Temporal Gyrus<br>Parahippocampal Gyrus<br>Entorhinal Cortex<br>Fusiform Gyrus
Occipital	Superior Occipital Gyrus<br>Middle Occipital Gyrus<br>Inferior Occipital Gyrus<br>Cuneus<br>Lingual Gyrus
Limbic	Rostral Anterior Cingulate Cortex<br>Subcallosal Anterior Cingulate Cortex<br>Subgenual Anterior Cingulate Cortex<br>Dorsal Anterior Cingulate Cortex

	Posterior Cingulate Cortex<br>Hippocampus
Insula	Insula
Amyg	Amygdala
Basal Gang	Caudate Nucleus<br>Putamen<br>Globus Pallidus<br>Nucleus Accumbens
Thalamus	Thalamus<br>Hypothalamus

Detailed breakdown of macro regions and their constituent ROIs used in the SuStaIn analysis. Cortical regions (Frontal, Parietal, Temporal, Occipital) were analyzed separately for left and right hemispheres. All other regions were averaged across hemispheres.

Supplementary Methods S4. SuStaIn Analysis Technical Details

S4.1 Data Preparation

Prior to the SuStaIn analysis, the normalized regional volumes were converted to z-scores with adjustments for covariates including age, sex, and total intracranial volume (TIV). This covariate-adjusted standardization was performed using a refined regression approach where control subjects served as the normative reference.

The adjustment procedure was as follows:

1. For each biomarker, a linear regression model was fitted using only the control group data, with the regional brain volume as the dependent variable and age, sex, and total intracranial volume (TIV) as independent variables.
2. This control-trained model was then applied to the entire dataset to calculate predicted values.
3. Residuals were computed for all subjects by subtracting the predicted values from the observed biomarker values.
4. These residuals were standardized based on the control group statistics:
5. z-score = (residual - mean of control residuals) / standard deviation of control residuals
6. To maintain a consistent direction of pathological change, biomarkers showing a decrease with disease progression were identified and inverted. Specifically, biomarkers where the overall mean was lower than the control group mean were multiplied by -1.

This refined covariate-adjustment approach ensures that all standardization is anchored to the control population, providing a more accurate reference point for detecting disease-specific abnormalities. The resulting z-scores represent deviations from the healthy control distribution after accounting for demographic and volumetric confounders, facilitating more precise identification of disease-related volumetric changes across different brain regions.

After adjustment, we confirmed that the control group had a mean z-score of approximately 0 and a standard deviation of 1 across all biomarkers, validating the normalization procedure.

S4.2 SuStaIn Algorithm Parameters

The key parameters used in the SuStaIn analysis were:

- Maximum number of subtypes to evaluate: 5
- Number of Markov Chain Monte Carlo (MCMC) iterations: 200,000

- Number of expectation-maximization (EM) algorithm start points: 100
- Z-score thresholds: 1, 2, and 3
- Maximum z-score value: 5

For computational efficiency, we enabled parallel processing for the multiple start points.

S4.3 SuStaIn Results Processing

We developed a refined approach to extract and analyze the SuStaIn algorithm outputs, focusing on the probability distributions of Z-scores across different stages for each brain region. Instead of using the traditional positional variance diagrams (PVDs), we directly extracted the probabilities of each region reaching specific Z-score thresholds at each disease stage.

For each subtype, we created a comprehensive matrix representation where rows corresponded to brain regions and columns to disease stages. This matrix contained the probability distributions of different Z-score ranges at each stage, allowing us to precisely track when specific brain regions reached clinically meaningful levels of abnormality.

S4.4 Linear Interpolation of Z-scores

For each subtype, we extracted the probabilities of each brain region reaching specific Z-score thresholds (Z-score 1, 2, and 3) at each disease stage from the SuStaIn algorithm's output. Using these probabilities, we identified the earliest stage at which each Z-score threshold was most likely to be reached for each brain region. Between these threshold stages, we linearly interpolated the values to ensure smooth transitions. This approach created a progression where each brain region's values advanced from 0 → 1 → 2 → 3 as the disease progressed, with intermediate stages showing gradual transitions between these levels of abnormality.

For visualization, we created two complementary representations using MATLAB R2024b (Mathworks). First, we developed 3D brain renderings showing the regional progression of atrophy from multiple viewpoints (lateral and medial views of both hemispheres). Second, we created matrix-based visualizations displaying all brain regions simultaneously across disease stages. Both visualizations used the same distinctive color mapping: no change (Z-score 0) in light gray, initial change (Z-score 1) in blue, moderate change (Z-score 2) in yellow-green, and advanced change (Z-score 3) in pink.

The 3D visualizations displayed brain regions from multiple angles across different disease stages, allowing for comprehensive spatial assessment of disease progression. The 2D heatmap matrices arranged brain regions on the vertical axis and disease stages on the

horizontal axis, providing a complete overview of all regions simultaneously.

In both visualization formats, each region's color intensity varied according to the interpolated Z-score value, with fractional values between integer Z-scores represented by proportional color blending. This dual approach allowed us to simultaneously analyze both spatial patterns (which regions are affected) and temporal sequences (when regions become abnormal) across different subtypes of schizophrenia.

S4.5 Model Selection and Validation

To determine the optimal number of subtypes, we evaluated models with varying numbers of subtypes (from 1 to 5) using multiple approaches. We analyzed the log-likelihood trace from the MCMC sampling process to assess convergence and model stability. For each model, we extracted and compared the maximum, mean, and final log-likelihood values across different numbers of subtypes.

For formal model selection, we computed the Bayesian Information Criterion (BIC) for each model[6], calculated as:

BIC = -2 × log-likelihood + number of parameters × log(sample size)

where the number of parameters was estimated based on the number of subtypes and biomarkers. We selected the model with the lowest BIC value, which optimally balances model fit and complexity while imposing a stronger penalty for model complexity compared to other criteria. This approach provides a more principled method for selecting the appropriate number of subtypes compared to heuristic approaches.

For the final characterization of disease subtypes and their progression patterns, we utilized the full dataset model with the optimal number of subtypes as determined by BIC. The uncertainty in the ordering of events within each subtype was estimated using MCMC sampling with 200,000 iterations. For visualization purposes, we generated positional variance diagrams showing the probability of each region reaching each z-score threshold at each stage of the disease progression.

S4.6 Individual Subject Classification

Each participant was assigned to a SuStaIn subtype and stage based on the maximum likelihood. The subtype assignment provided information about the most likely pattern of brain atrophy, while the stage assignment indicated the position along the progression pattern, serving as a proxy for disease severity or progression. Subtype assignment for each individual was determined by calculating the probability of belonging to each subtype, with assignment made to the subtype with the highest probability.

Supplementary Figure S5: Flow of participant selection.

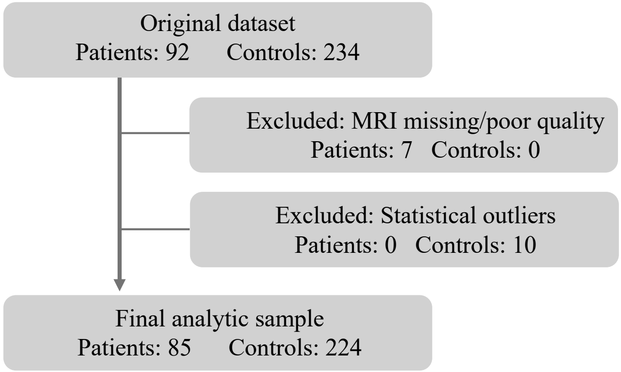

The original dataset included 92 patients with schizophrenia and 234 healthy controls. Seven patients were excluded due to missing MRI data or severe motion artifacts. In addition, 10 healthy controls were excluded as statistical outliers based on age- and sex-adjusted volumetric profiles using multivariate outlier detection (Isolation Forest algorithm). The final analytic sample comprised 85 patients and 224 healthy controls.

Supplementary Table S6. Demographic and Clinical Characteristics by Subtype

Characteristic	Subtype0 (n=55)	Subtype1 (n=30)	p value
Sample size	55	30	
Age years	39.3 ± 9.6	40.5 ± 11.9	0.938
Sex n (%)			0.024
Male	23 (41.8)	21 (70.0)	
Female	32 (58.2)	9 (30.0)	
Handedness n (%)			1.000
Right	51 (92.7)	28 (93.3)	
Left/Other	4 (7.3)	2 (6.7)	
Scanner type n (%)			0.429
Trio	32 (58.2)	14 (46.7)	
Tim Trio	23 (41.8)	16 (53.3)	
Disease stage	2.04 ± 2.11	2.60 ± 2.69	0.582
Illness duration years	15.3 ± 8.4	13.5 ± 11.3	0.104
Antipsychotic dose CPZ eq. mg/day	652.3 ± 504.8	488.1 ± 321.5	0.347

Comparison of demographic, scanner, and clinical characteristics between Subtype0 (anterior-onset, n=55) and Subtype1 (posterior-onset, n=30). Data are presented as mean ± SD or n (%). Statistical comparisons were performed using Mann-Whitney U tests for continuous variables and chi-square tests for categorical variables. CPZ eq. = chlorpromazine equivalents.

Supplementary Table S7. Associations Between Disease Stage and Medication Variables

A. Correlations between disease stage and continuous medication variables

Variable	Spearman's rho	p value	n
**Overall correlations**			
Illness duration	-0.035	0.752	84
Antipsychotic dose (CPZ eq.)	0.070	0.527	84
**Subtype 0 correlations**			
Illness duration	0.035	0.804	54
Antipsychotic dose (CPZ eq.)	0.208	0.132	54
**Subtype 1 correlations**			
Illness duration	-0.132	0.486	30
Antipsychotic dose (CPZ eq.)	-0.239	0.203	30

B. Disease stage by concomitant medication use

Concomitant medication	No use	Use	p value
Anticholinergic	2.47 ± 2.49 (n=49)	1.97 ± 2.08 (n=35)	0.450
Benzodiazepine	1.94 ± 1.73 (n=31)	2.45 ± 2.61 (n=53)	0.769
Mood stabilizer	2.25 ± 2.36 (n=73)	2.36 ± 2.20 (n=11)	0.818
Antidepressant	2.15 ± 2.22 (n=73)	3.00 ± 2.97 (n=11)	0.382

Correlations between SuStaIn-derived disease stage and medication variables. Part A shows Spearman's rank correlations for illness duration and antipsychotic dose (chlorpromazine equivalents) in the overall patient sample and by subtype. Part B shows disease stage (mean ± SD) stratified by concomitant medication use, with p-values from Mann-Whitney U tests. No significant associations were observed between disease stage and any medication variable (all $p > 0.05$).

Supplementary Table S8: Structure-Stage Associations in Subtype

	Regions	ρ	p-value	FDR q-value
Subtype 0	Precuneus_R	-0.482	0.0004	0.0264
	Dorsal Anterior Cingulate Cortex_R	-0.449	0.0006	0.0264
	Rectus Gyrus_R	-0.421	0.0013	0.0278
	Dorsal Anterior Cingulate Cortex_L	-0.432	0.0014	0.0278
	Supramarginal Gyrus_L	-0.431	0.0015	0.0278
	Rectus Gyrus_L	-0.407	0.0020	0.0312
	Middle Frontal Gyrus_R	-0.384	0.0034	0.0438
	Middle Frontal Gyrus_L	-0.383	0.0037	0.0438
	Superior Frontal Gyrus_pole_R	-0.368	0.0049	0.0473
	Posterior Cingulate Cortex_R	-0.372	0.0054	0.0473
	Precuneus_L	-0.373	0.0060	0.0473
	Angular Gyrus_R	-0.369	0.0062	0.0473
	Parahippocampal Gyrus_L	-0.367	0.0064	0.0473
	Superior Parietal Gyrus_R	-0.360	0.0073	0.0477
	Inferior Occipital Gyrus_R	-0.353	0.0075	0.0477
	Inferior Frontal Gyrus Orbitalis_L	-0.355	0.0081	0.0483
Subtype 1	Cuneus_R	-0.539	0.0023	0.223
	Lingual Gyrus_R	-0.467	0.0089	0.432
	Posterior Basal Forebrain_L	0.444	0.0144	0.467
	Middle Occipital Gyrus_R	-0.386	0.0354	0.682
	Dorsal Anterior Cingulate Cortex_R	-0.373	0.0432	0.682

ρ, Spearman's partial correlation coefficient; FDR, False Discovery Rate; L, left; R, right.

Partial correlations between regional brain volumes and disease progression stage for both subtypes, controlling for age, sex, handedness, and total intracranial volume. For Subtype0, 16 regions showed significant associations after FDR correction ($q < 0.05$). For Subtype1, 5 regions showed associations based on uncorrected p-values ($p < 0.05$), though none survived FDR correction for multiple comparisons.

Supplementary Figure S9: Partial correlations between disease progression stage and regional brain volumes in Subtype0 after covariate adjustment

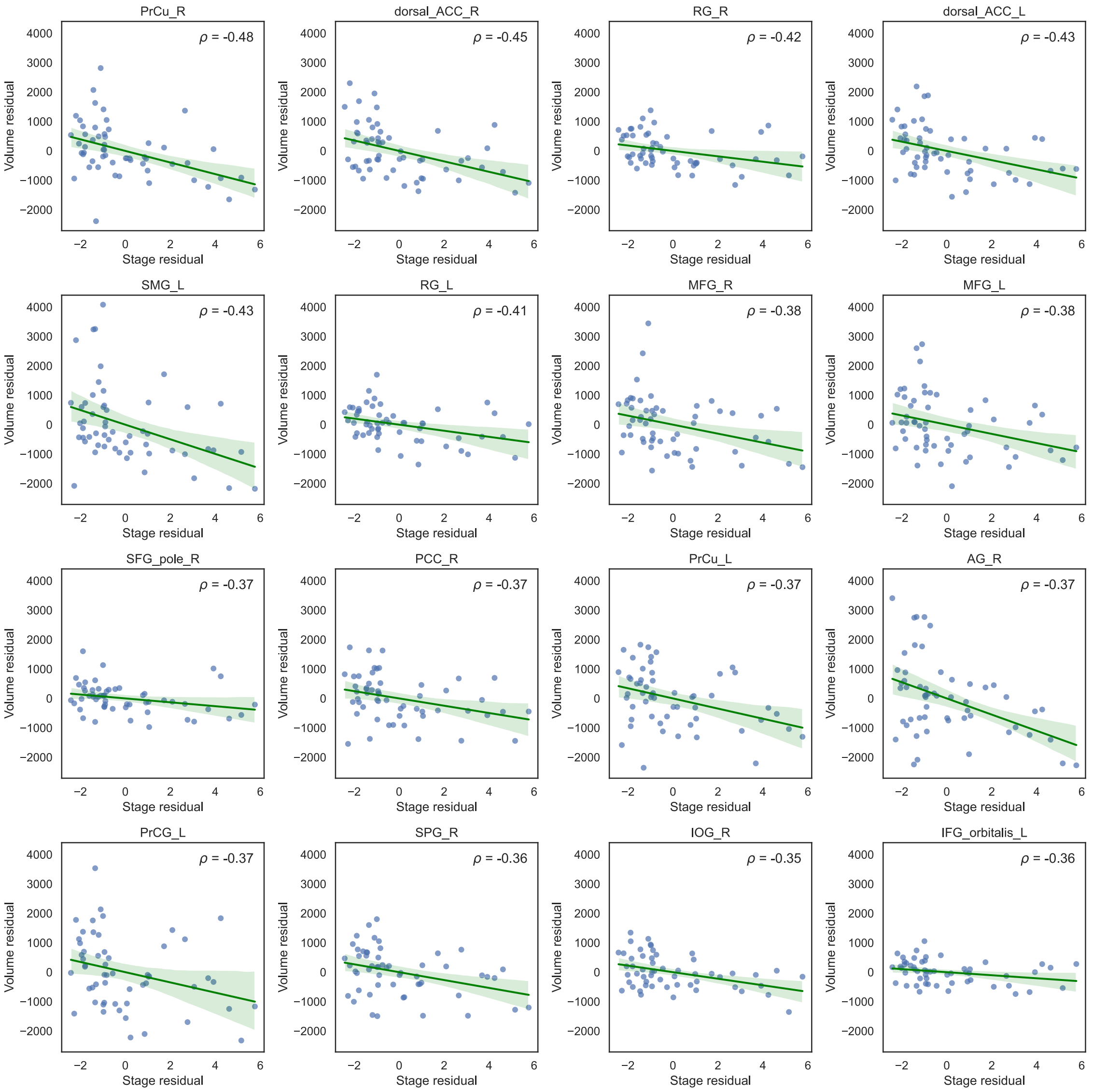

Scatter plots showing the relationships between residualized disease progression stage (x-axis) and residualized regional brain volumes (y-axis) for the 16 brain regions that demonstrated significant negative correlations after FDR correction ($q < 0.05$) in Subtype0. Both variables have been residualized against covariates (age, sex, handedness, and total intracranial volume) using linear regression. Each panel displays individual data points with fitted regression lines (green) and 95% confidence intervals (shaded areas). Spearman's partial correlation coefficients (ρ) are displayed in the top right of each panel.

Abbreviations: PrCu = precuneus; dorsal_ACC = dorsal anterior cingulate cortex; RG =

rectus gyrus; SMG = supramarginal gyrus; MFG = middle frontal gyrus; SFG_pole = superior frontal gyrus pole; PCC = posterior cingulate cortex; AG = angular gyrus; PrCG = parahippocampal gyrus; SPG = superior parietal gyrus; IOG = inferior occipital gyrus; IFG_orbitalis = inferior frontal gyrus orbitalis; L = left; R = right.

Supplementary Figure S10: Partial correlations between disease progression stage and regional brain volumes in Subtype1 after covariate adjustment

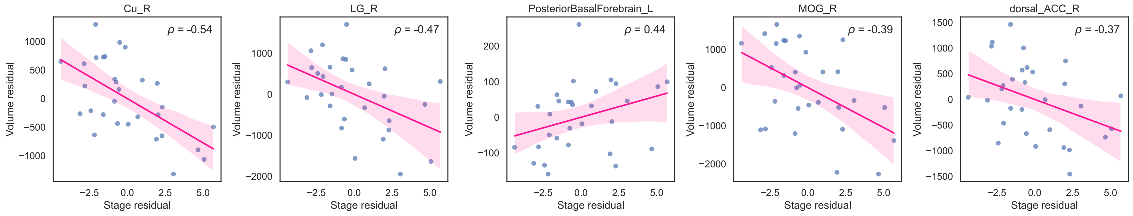

Scatter plots demonstrate the relationship between stage residuals (x-axis) and volume residuals (y-axis) after controlling for confounding variables. Each panel represents one brain region, with partial correlation coefficients (ρ) displayed in the upper right corner. Pink regression lines with confidence intervals indicate the linear trend and uncertainty.

Brain region abbreviations: Cu_R, cuneus right; LG_R, lingual gyrus right; PosteriorBasalForebrain_L, posterior basal forebrain left; MOG_R, middle occipital gyrus right; dorsal_ACC_R, dorsal anterior cingulate cortex right. ρ represents the adjusted partial correlation coefficient.

Supplementary Table S11: Functional connectivity correlations with disease progression stage in Subtype0

Connection	ρ	p	FDR q	FWER	scanner p
Angular Gyrus_R – Entorhinal Cortex_L	-0.565	< 0.0001	0.228	*	0.473
Postcentral Gyrus_L – Inferior Temporal Gyrus_L	-0.460	< 0.001	0.996	ns	0.217

Partial Spearman correlations between brain network edge connectivity and disease stage, adjusted for age, sex, handedness, and scanner. Only connections with $p < 0.001$ are shown. ρ: partial Spearman correlation coefficient; p: permutation test p-value (20,000 permutations); FDR q: false discovery rate-corrected q-value; FWER: family-wise error rate correction (: significant, ns: not significant); scanner p: one-way ANOVA p-value testing for scanner effects across sites.*

Supplementary Table S12: Functional connectivity correlations with disease progression stage in Subtype1

Connection	ρ	p	FDR q	scanner p
Right Supramarginal Gyrus – Right Mammillary	0.662	< 0.0001	0.076	0.342
Right Lingual Gyrus – Right Insula	0.699	< 0.0001	0.076	0.337
Left Lingual Gyrus – Right Insula	0.704	< 0.0001	0.076	0.894
Right Superior Parietal Gyrus – Left PosteriorBasalForebrain	0.674	< 0.0001	0.076	0.638
Left Parahippocampal Gyrus – Right AnteriorBasalForebrain	0.692	< 0.0001	0.076	0.993
Left Parahippocampal Gyrus – Right NucAccumbens	0.673	< 0.0001	0.076	0.488
Right Postcentral Gyrus – Left Insula	0.643	< 0.001	0.098	0.612
Right Superior Parietal Gyrus – Left subcallosal_ACC	0.643	< 0.001	0.171	0.693
Left Superior Frontal Gyrus – Right Putamen	-0.615	< 0.001	0.282	0.297
Left Superior Temporal Gyrus_pole – Left AnteriorBasalForebrain	0.602	< 0.001	0.282	0.335
Right Supramarginal Gyrus – Left Mammillary	0.581	< 0.001	0.282	0.440
Left Posterior Cingulate Cortex – Right Globus Pallidus	-0.582	< 0.001	0.282	0.106
Right Middle Temporal Gyrus – Left Hippocampus	-0.582	< 0.001	0.282	0.668
Left Parahippocampal Gyrus – Left NucAccumbens	0.588	< 0.001	0.282	0.784

Partial Spearman correlations between brain network edge connectivity and subtype1, adjusted for age, sex, handedness, and scanner. Only connections with $p < 0.001$ are shown. ρ: partial Spearman correlation coefficient; p: permutation test p-value (20,000 permutations); FDR q: false discovery rate-corrected q-value; scanner p: one-way ANOVA p-value testing for scanner effects across sites. None of the connections survived family-wise error rate (FWER) correction.

Reference

1. Nishimaki K, Onda K, Ikuta K, et al. OpenMAP-T1: A Rapid Deep-Learning Approach to Parcellate 280 Anatomical Regions to Cover the Whole Brain. *Hum Brain Mapp*. 2024;45(16):e70063. doi:10.1002/hbm.70063

2. Tustison NJ, Avants BB, Cook PA, et al. N4ITK: Improved N3 Bias Correction. *IEEE Trans Med Imaging*. 2010;29(6):1310-1320. doi:10.1109/TMI.2010.2046908

3. Tzourio-Mazoyer N, Landeau B, Papathanassiou D, et al. Automated Anatomical Labeling of Activations in SPM Using a Macroscopic Anatomical Parcellation of the MNI MRI Single-Subject Brain. *NeuroImage*. 2002;15(1):273-289. doi:10.1006/nimg.2001.0978

4. Avesta A, Hossain S, Lin M, Aboian M, Krumholz HM, Aneja S. Comparing 3D, 2.5D, and 2D Approaches to Brain Image Auto-Segmentation. *Bioengineering (Basel)*. 2023;10(2):181. doi:10.3390/bioengineering10020181

5. Oishi K, Faria A, Jiang H, et al. Atlas-based whole brain white matter analysis using large deformation diffeomorphic metric mapping: Application to normal elderly and Alzheimer's disease participants. *Neuroimage*. 2009;46(2):486-499. doi:10.1016/j.neuroimage.2009.01.002

6. Burnham KP, Anderson DR. *Model Selection and Multi-Model Inference: A Practical Information-Theoretic Approach*. New ed. Springer; 2011.
